# Knowledge, practices and perceived barriers of physiotherapists involved in disaster management: a cross-sectional survey of Nigeria-based and trained physiotherapists

**DOI:** 10.1093/inthealth/ihab019

**Published:** 2021-04-15

**Authors:** Chidiebele P Ojukwu, Obumneke G Eze, Ekezie M Uduonu, Adaora J Okemuo, Joseph O Umunnah, Stephen S Ede, Chioma L Onuchukwu, Emelie M Anekwu, Anne U Ezeigwe

**Affiliations:** Department of Medical Rehabilitation, Faculty of Health Sciences and Technology, University of Nigeria, Enugu, Nigeria, 400241; Department of Medical Rehabilitation, Faculty of Health Sciences and Technology, University of Nigeria, Enugu, Nigeria, 400241; Department of Medical Rehabilitation, Faculty of Health Sciences and Technology, University of Nigeria, Enugu, Nigeria, 400241; Department of Medical Rehabilitation, Faculty of Health Sciences and Technology, University of Nigeria, Enugu, Nigeria, 400241; Department of Medical Rehabilitation, Faculty of Health Sciences and Technology, Nnamdi Azikiwe University, Nnewi Campus, Nigeria, 420007; Department of Medical Rehabilitation, Faculty of Health Sciences and Technology, University of Nigeria, Enugu, Nigeria, 400241; Department of Gerontology, Faculty of Social Sciences, University of Southampton, Southampton, UK, S017 1BJ; Department of Physiotherapy, Enugu State University Teaching Hospital, Parklane, Enugu State, Nigeria, P.M.B 1030; Department of Physiotherapy, Federal Teaching Hospital, Abakaliki, Ebonyi State, Nigeria, 480241; Department of Physiotherapy, Enugu State University Teaching Hospital, Parklane, Enugu State, Nigeria, P.M.B 1030

**Keywords:** barriers, disaster management, knowledge, Nigeria, physiotherapy, practice

## Abstract

**Background:**

Disasters represent substantial health risks to the human population. Proper interventions at all stages of disaster management (DM) are essential for human-related outcomes. The role of physiotherapy in DM should not be underestimated, but unfortunately information on the involvement of physiotherapists in DM is limited in sub-Saharan Africa.

**Methods:**

One hundred and fifty Nigeria-based and trained physiotherapists were recruited to complete a questionnaire investigating the knowledge, practices and perceived barriers of the role of physiotherapists in DM.

**Results:**

Physiotherapists had moderate knowledge of their role at every stage of DM. However, their involvement in specific DM-related activities during various stages of DM in the country was low. Major barriers to the involvement of physiotherapists in DM included a lack of established government policies on the integration of physiotherapists in DM (90.0%) and a lack of specialty clinical practice areas in DM (89.3%). For improved involvement of physiotherapists in DM in Nigeria, respondents recommended creating an awareness of the role of physiotherapists in DM (91.3%), providing continuous education programmes on DM (90.6%) and inclusion of DM in physiotherapy training programmes in Nigeria (90.0%).

**Conclusion:**

Physiotherapists perceived that they are not adequately involved in DM in Nigeria, despite their moderate knowledge of their role during DM.

## Introduction

Exposure to risk is an integral part of human existence and, with the rapid advancement in science and technology, the factors precipitating risk to individuals are increasingly associated with various forms of disasters. Disasters are events and situations that overwhelm local capacity and thus require external assistance from national and international bodies.^1^ These events are sudden and calamitous and they seriously disrupt the functioning of a community or society, causing human, material and economic or environmental losses that exceed the community's or society's ability to cope using its own resources.^2^

Natural disasters include floods, hurricanes, tornadoes, volcanic eruptions and earthquakes.^3^ They commonly result in loss of life or damage to property and typically leave economic damage in their wake, the severity of which depends on the affected population's resilience or ability to recover, as well as the available infrastructure.^3^ On the other hand, human-induced disasters occur as a result of identifiable human actions, deliberate or otherwise. Apart from technological and ecological disasters, this mainly involves situations in which civilian populations experience trauma, casualties, the loss of property, an impediment of basic services and their means of livelihood (e.g. because of war or civil strife).^4^

Disasters commonly occur in poorly resourced regions where disaster response plans do not include strategies for physical rehabilitation or specifically account for people with new or pre-existing disabilities.^2^ The vulnerable populations are mostly affected by a disaster, leaving them with several challenges. Large-scale disasters result in significant numbers of disabling impairments, including spinal cord injuries, traumatic brain injuries, limb amputations, long bone fractures and peripheral nerve injuries.^5^

Disaster management (DM) is a continuum of four distinct stages, consisting of prevention, preparation and planning, immediate response and recovery.^6^ Each of these stages requires multidisciplinary approaches, ranging from relevant medical to non-medical interventions. Medical approaches to DM should involve the indispensable role of physiotherapists as part of the multidisciplinary health team. According to the World Confederation of Physical Therapy (WCPT),^6^ the role of physiotherapists in emergency response is evolving, with increased recognition internally and externally regarding the importance of rehabilitation in a range of humanitarian settings. The expertise of physiotherapists in disability and functioning places them in an appropriate position in DM to assist with humanitarian aid, particularly during the rehabilitation phase of injured individuals. Considering the disabling impairments associated with disasters,^6–8^ physiotherapists are increasingly being integrated into emergency management teams.^9,10^ However, Waldrop^11^ observed that despite the involvement of physiotherapists in DM in the USA, their role was not clearly defined.

In the USA, a guide that describes the role of physiotherapists in disaster response was published in 2004,^12^ and in 2016 a more detailed global report was developed by the WCPT.^6^ According to this report, when disasters occur, the functions of a physiotherapist in the immediate response stage include: assessment of the general need for rehabilitation in the disaster situation; mapping available rehabilitation and other specialist services for those with injuries and/or disabilities; providing acute rehabilitation, including orthopaedic, neurological, respiratory and burns rehabilitation, in local hospitals, the community or as part of a non-government organisation or emergency medical team; providing holistic education for patients, caregivers and other health personnel; sorting the management of and/or referring survivors; co-coordinating discharge, referral and follow-up; assessing, prescribing, fitting and providing assistive devices and providing training in their use and maintenance; providing musculoskeletal rehabilitation or manual handling training and support to other professionals involved in the response; and training rehabilitation colleagues and community workers on necessary skills. During the recovery stages of DM, the functions of a physiotherapist include: continuity of care; advocating for and advising around inclusive reconstructions; building the capacity of local services to respond both to current increased need and future disasters; getting involved in multidisciplinary task forces that prepare long-term rehabilitation services; and supporting those with existing and new disabilities.

Despite these recommendations and guidelines, some studies^6,13^ have considered the involvement of physiotherapists in DM as insufficient for global impact. Harrison^1^ revealed that physiotherapists who participated in DM in the USA felt underprepared, considering that they were not originally factored into DM teams. During the times that their services were utilised, their role included patient care and organising physiotherapy and other services. In 2017, Trivedi and Rathod^2^ undertook a survey to determine the roles of physiotherapists who participated in DM during the 2004 tsunami in South India as well as the 2001 earthquake in western India. The results of the study indicated that physiotherapists were not involved in the preparatory phase but were involved in wound care, assessment and treatment of disabilities during the response and recovery stages of DM. Mulligan et al.^13^ reported that physiotherapists who participated in the February 2011 earthquake in Canterbury offered psychological support to the survivors, in addition to physical care. Nepal's Physiotherapy Association^14^ also shared that physiotherapists’ roles in the 2015 Nepal earthquake included assisting in the screening and transfer of patients, as well as providing acute injury management alongside other healthcare providers.

In sub-Saharan Africa, the role of physiotherapists in DM has yet to be explored. To date, there is a paucity of documented guidelines and recommendations specifying the roles of Nigeria-based physiotherapists in DM. Surprisingly, the inclusion of DM as a curricular requirement in physiotherapy training has yet to be implemented in Nigeria. It is noteworthy that Nigeria has been reported as the most populous black nation that has previously faced natural and manmade disasters^4^ and yet there are no substantial records of physiotherapy involvement at the various phases of DM. To initiate the inclusion of physiotherapists in DM education as well as to implement DM education in physiotherapy training programmes in Nigeria, a need was identified to investigate the knowledge, practices and perceived barriers regarding the role of physiotherapists in DM among Nigeria-based and trained physiotherapists. This preliminary study will create opportunities for improvement in further related studies, as well as stimulating interest and activities towards the development of physiotherapy practice guidelines for DM in Nigeria.

## Methods

### Study participants

A sample of 150 registered physiotherapists with at least 1 y of work experience participated in this cross-sectional descriptive study that was conducted during April–June 2019. Only those physiotherapists who were trained in and were practising in Nigeria during the time of the study were considered eligible for inclusion.

### Study instrument

A self-developed questionnaire, structured from the contents of the WCPT DM report,^6^ was used to obtain information from physiotherapists. The four-sectioned structured questionnaire contained both open- and close-ended questions and was developed in English. The first section (A) of the instrument investigated physiotherapists’ personal and occupational characteristics. In subsequent sections (B–D), the knowledge and attitudes of physiotherapists on their specific roles regarding preparation and planning during the immediate response and recovery phases of DM were assessed. The specific roles assessed by the questionnaire were adopted from the WCPT^6^ report on the role of physical therapists in DM. In addition, the instrument assessed the barriers perceived by physiotherapists to their involvement in DM in Nigeria.

The questionnaire was reviewed for content and face validity by four experts (two clinical and two academic physiotherapists). Subsequent to the implementation of necessary corrections, a test-retest reliability procedure was conducted among 10 physiotherapists. The questionnaire was administered twice within a 4-wk test interval. Intraclass correlation analysis for internal consistency at 95% CI yielded a correlation coefficient of r=0.896 (p=0.001), indicating very high agreement of the results.^15^

### Procedures for data collection

Data collection was performed via two means: (1) electronic survey platform. Survey questions were entered into a Google form and the website link was circulated via email and social media platforms (Whatsapp, telegram and Facebook) that housed Nigerian physiotherapists. Responses were saved directly onto Google cloud; and (2) a few copies of the questionnaire in paper form were administered to about 50 physiotherapists within proximity areas who had poor or no access to internet facilities. The physiotherapists filled out the forms by themselves under the guidance of researchers or assistants and the obtained data were manually processed. Subsequently, all data were collated and stored in Microsoft Excel spreadsheets before being transferred to SPSS for analyses. To avoid multiple entries from a respondent, Google Apps Script was used to validate and delete duplicate data, after which data from 179 forms remained. However, only 150 of the 179 forms were completely filled with missing data handled during the data analysis stage. Stored data were saved in the cloud and detachable hard disks with a confidential password were made available to the research collaborators. So far, the duration of the stored data is until infinity, unless the authors agree otherwise in future.

### Data analysis

Participants’ knowledge was graded as Yes or No with numerical ratings of 1 or 2, respectively. A score of 1 or 0 represented either good or poor knowledge, respectively, of a specific variable. Responses were classified as good (80–100%), moderate/fair (60–79%) or poor (<60%).^16^

Descriptive statistics of frequency counts and percentages were used to summarise data. Missing data were handled through Listwise or case deletion, and only fully completed questionnaires were considered for analysis. Data analysis was performed with the Statistical Package for Social Sciences version 21 (IBM SPSS Statistics for Windows, version 21.0. Armonk, NY: IBM Corp.).

## Results

The general characteristics of the respondents (Table 1) shows that the majority of participants were female (52.0%), within an age range of 20–45 y (84.7%), worked in federal hospitals/centres (67.3%), had >5 y of work experience and bachelor degrees as their highest academic qualification.

**Table 1.** General characteristics of the respondents

Variable	Frequency	%
Age, y		
<20	1	0.6
20–45	127	84.7
>45	22	14.7
Gender		
Male	72	48.0
Female	78	52.0
Educational level		
Bachelor's degree	121	80.7
Master's degree	22	14.7
Doctorate degree	7	4.6
Work organisation		
Federal institution	101	67.3
State institution	17	11.3
Private practice	22	14.6
Job experience, y		
≤5	89	59.3
>5	61	40.7

From a general perspective, 68.7% of the physiotherapists acknowledged their potential role in DM, with more knowledge expressed regarding their role in disaster recovery (84.0%) compared with other stages of DM (Table 2). Most of them (86.7%) were aware of the disaster-related hazards facing Nigeria.

**Table 2.** Knowledge of physiotherapists’ roles in disaster management

	Yes	No
Variables	n (%)	n (%)
General roles in disaster management	103 (68.7)	47 (31.3)
Roles of physiotherapy involvement in different stages of disastermanagement		
Roles in disaster prevention	106 (70.7)	44 (29.3)
Roles in preparation and planning of disasters	84 (56.0)	66 (44.0)
Roles in disaster response	97 (64.7)	53 (35.3)
Roles in disaster recovery	126 (84.0)	24 (16.0)

Investigations of DM-related activities that have been integrated into physiotherapy practice in Nigeria showed that several areas have not been adequately explored. Creating awareness of physical and rehabilitation needs (38.8%) as well as the consequences of disasters (diseases, injuries, psychological and social impacts) were reported as the most common DM-related activities integrated into Nigerian physiotherapy practice (Table 3).

**Table 3.** Reports on disaster management (DM)-related activities that have been integrated into physiotherapy practice in Nigeria

	Yes	No
Activities	n (%)	n (%)
Awareness creation on possible risks of disaster	31 (20.7)	119 (79.3)
Awareness creation on consequences of disasters (diseases, injuries, psychological and social impacts)	52 (34.7)	98 (65.3)
Awareness creation on physical and rehabilitation needs	58 (38.8)	92 (61.2)
Lobbying governmental, non-governmental organisations and institution to be prepared for disasters and inclusion of physiotherapist in planning.	28 (18.7)	122 (81.3)
Ensuring physiotherapy participation in DM planning and implementation	31 (20.7)	119 (79.3)
Training physiotherapists on DM	41 (27.3)	109 (72.7)
Interprofessional collaboration on DM	38 (25.3)	112 (74.7)
Development of clinical specialty on DM	38 (25.3)	112 (74.7)

At the planning stage of DM, Table 4 shows that respondents displayed knowledge regarding their role in contributing to the development of DM plans (81.3%) compared with their role in contributing to planning for emergencies in their place of practice or locality (17.3%). However, their involvement in performing these roles was limited.

**Table 4.** Respondents’ knowledge of and involvement in the disaster management planning stage

	Knowledge	Involvement
Variable	n (%)	n (%)
Contributions to development of disaster management plans	122 (81.3)	8 (5.0)
Contribution to planning for emergencies in their place of practice or locality.	26 (17.3)	6 (3.5)

At the disaster response stages (Table 5), the majority of respondents displayed more knowledge regarding their roles in providing acute rehabilitation for the injured (94.0%), preventive care for vulnerable people (92.0%), mapping available rehabilitation and other specialist services for the injured (91.3%), as well as assessing, prescribing and fitting assistive devices (91.3%). Involvement in these stages and others was minimal. The majority of responses indicated that only 10 of the participants (6.7%) were involved in providing acute rehabilitation for the injured and educating patients, caregivers and other health personnel during the response stage of DM.

**Table 5.** Respondents’ knowledge of and involvement in specific activities at disaster response stages

	Knowledge	Involvement
Variable	n (%)	n (%)
Mapping available rehabilitation and other specialist services for the injured	137 (91.3)	7 (4.7)
Provision of acute rehabilitation for the injured	141 (94.0)	10 (6.7)
Educating patients, caregivers and other health personnel	135 (90.0)	10 (6.7)
Sorting, management and/or referring survivors	111 (74.0)	5 (3.3)
Coordinating discharge, referral and follow-up	112 (74.7)	4 (2.7)
Providing psychosocial support or referral to appropriate	116 (77.3)	7 (4.7)
Assessment, prescription and fitting of assistive devices	137 (91.3)	8 (5.3)
Environmental assessment and adaptation to ensure accessibility for the injured and disabled	128 (85.3)	4 (2.7)
Identifying individuals at risk of more injury	137 (91.3)	7 (4.7)
Provision of preventive care for vulnerable people	138 (92.0)	6 (4.0)

The knowledge of participants regarding specific activities during the recovery stage of DM (Table 6) showed that the majority were aware of their role in supporting individuals with existing and new disabilities (92.7%) and getting involved in multidisciplinary task forces that prepare long-term rehabilitation services (92.0%). Getting involved in multidisciplinary task forces that prepare long-term rehabilitation services was identified as the activity that the majority (7.3%) of participants was involved in at this stage of DM.

**Table 6.** Respondents’ knowledge of and involvement in disaster recovery stage

	Knowledge	Involvement
Variable	n (%)	n (%)
Linking the acute response to ongoing rehabilitation and support (continuity of care)	137 (91.3)	10 (6.7)
Advocating for and advising around inclusive reconstructions	116 (77.3)	3 (2.0)
Building the capacity of local services to respond both to current increased need and future disasters	123 (82.0)	5 (3.3)
Getting involved in multidisciplinary task forces that prepare long-term rehabilitation services	138 (92.0)	11 (7.3)
Supporting those with existing and new disabilities	139 (92.7)	10 (6.7)

The major barriers identified to the involvement of physiotherapists in DM were a lack of established government policies on the integration of physiotherapists into DM (90.0%) and a lack of specialty clinical practice areas in DM (89.3%). The majority of participants recommended creating awareness regarding the role of physiotherapists in DM among the public and other healthcare professionals (91.3%), and providing continuous education programmes on DM (90.7%) as a means to improving the knowledge and involvement of physiotherapists in DM (Table 7).

**Table 7.** Perceived barriers of physiotherapy involvement in disaster management and recommendations for improvement

Variable	Frequency (%)
Barriers	
Lack of established government policies on the integration of physiotherapists in disaster management	135 (90.0)
Lack of specialty clinical practice areas in disaster management	134 (89.3)
None or lack of awareness of the roles of physiotherapists in disaster management among the public and other healthcare professionals	128 (85.3)
Lack of continuing education programmes (e.g seminars, workshops) on disaster management	119 (79.3)
Non-inclusion of disaster management in the curriculum of physiotherapy in Nigeria	118 (78.6)
Recommendations	
Creating an awareness of the roles of physiotherapists in disaster management among the public and other healthcare professionals	137 (91.3)
Provision of continuing education programmes on disaster management.	136 (90.7)
Inclusion of disaster management in physiotherapy training programmes in Nigeria	135 (90.0)
Establishment of government policies on the integration of physiotherapists in disaster management	134 (89.3)
Facilitation of specialty clinical practice areas in disaster management	134 (89.0)

## Discussion

Nigeria is burdened with major disaster scenarios, including floods, fires, collapsed buildings, road traffic accidents, epidemics, maritime-related disasters, oil spills, aviation disasters and rail accidents.^17^ DM is an ongoing activity in the country through various departments and units of the National Emergency Management Agency (NEMA).^18^ This novel study investigated the knowledge and involvement of physiotherapists in Nigeria's DM activities.

Generally, participants in this study had moderate knowledge regarding their role in DM with varying knowledge levels during the four stages of DM, namely, prevention, preparation/planning, response and recovery. The participants had more awareness regarding their roles during the recovery stage compared with other stages. This was not surprising as the roles of physiotherapists in Nigeria are predominantly focused on rehabilitation, unlike what is required in the prevention and acute management of diseases. More detailed findings regarding the participants’ knowledge and involvement during the different stages of DM are discussed below.

### Disaster preparation and planning

During this first stage of DM, physiotherapists displayed more knowledge regarding their role in developing DM plans compared with planning for emergencies in their place of practice or locality. These two components of disaster planning are very important and require the active involvement of physiotherapists. However, this study showed that the involvement of physiotherapy during the first stage of DM in Nigeria is negligible. Studies from other climes^1,2^ have shown that physiotherapists from other areas were not involved at this stage of DM either.

Preparation and planning for disasters requires building the necessary capacity to efficiently manage all types of emergency and achieve orderly transition from response through to the recovery stage.^6^ Significantly, this capacity includes human, financial, technological and infrastructural resources. To this end, physiotherapists should be involved in these planning processes, including procurement and supply of equipment, training activities, developing strategic plans for coordination, evacuation and public information.^19^ These planning strategies are restricted in Nigerian DM processes by limited financial, equipment, accommodation and mobilising resources.^20^ In addition, personnel inadequacies in the Nigerian healthcare system have made the scenario of not being prepared for DM worse.^21^

Considering that major disasters in Nigeria occur mainly in the rural areas,^21^ which are medically supported by the primary healthcare sector, physiotherapists need to be involved in the delivery of primary healthcare in Nigeria. This has not been the case, making it unrealistic for them to be fully involved in disaster planning activities at the community level. The prime function of NEMA is disaster preparedness and mitigation activities.^18^ Non-inclusion of physiotherapists in the execution of this role suggests major deficiencies in DM activities, as a team response with all healthcare professionals is pertinent to efficient DM outcomes.^14^

### Disaster response stage

During the disaster response stage of DM, the role of physiotherapists includes co-coordinating care, assessment, provision of acute rehabilitation, health education preventive care and appropriate referral.^6^ In the current study, physiotherapists were knowledgeable about their specific role at this stage of DM, particularly regarding the provision of acute rehabilitation for the injured. However, their knowledge was not matched to their levels of involvement in the implementation of these functions. In other countries, physiotherapists who had participated in the response stages of DM reported that their roles were poorly defined.^1,2,14^ It is clear that in the first few days of disaster response, the confusion as events unfold may precipitate an unstructured approach of physiotherapy services that progressively aligns better during subsequent days.

The response stage of DM is critical, considering the nature of casualties that arise from disasters.^6^ In Nigeria, the immediate effects of disasters include musculoskeletal trauma, burns and physical disabilities, which all require the acute intervention of physiotherapists for effective human outcomes. It is possible that some of the disaster-related conditions finally managed by physiotherapists will have occurred during the chaotic events that take place during the response stage. For instance, poor knowledge of lifting techniques by a disaster responder may result in severe injuries with neurological implications during transfers from accident scenes.

Evaluation and assessment of disaster damage, which is a major function of NEMA,^18^ cannot be achieved effectively without an assessment of the health implications of a disaster. This role, which is the sole responsibility of the healthcare system, can best be achieved by integrating the activities of all healthcare professions, including orthopaedic and trauma surgeons, physicians, physiotherapists, radiographers, nurses and pharmacists.

### Disaster recovery stage

Physiotherapists in this study demonstrated good knowledge regarding their specific roles during the last stage of DM. The participants were more aware of their role in supporting existing and new disabilities as well as continuity of care. Typically, physiotherapists are referred to as rehabilitation specialists in Nigeria, a perception that has limited their role in healthcare delivery to the recovery stage of most conditions. Despite this, perception of their involvement during the recovery stage of DM was poor, as revealed by the current study's findings. In other countries, physiotherapists have been involved in long-term rehabilitation of patients^1,2,14^ during this stage of DM. It is noteworthy that, during this stage, the care of people with disabilities due to injuries, poor basic surgical and medical care, mental health and emergency problems, is a necessity.^22^ In addition, the restoration and improvement of facilities and physical living conditions, as well as minimising disaster risk factors, are equally necessary.^6^ These roles and other rehabilitative services offered by physiotherapists are often not utilised in low- and middle-income countries,^23^ including Nigeria.

### Challenges of and recommendations for physiotherapy involvement in Nigerian DM

This study elucidates that the major challenge facing the involvement of physiotherapists in DM is the lack of established government policies on the integration of physiotherapists into DM. Neglecting to include physiotherapists in the various departments (in particular, relief and rehabilitation) and the 57 response units of NEMA^23^ will continue to hamper the efforts of physiotherapists to achieve their specific role in DM.

The regulatory board of physiotherapists in Nigeria has yet to include DM as a physiotherapy specialty area or to emphasise DM inclusion in the academic training curriculum of physiotherapy in Nigeria. These gaps in physiotherapy training and practice in the country have a huge impact on the interprofessional and public awareness of the role of physiotherapy in DM, which poses a further barrier to the inclusion of physiotherapists in DM teams. Participants in this study also attested to the limited efforts and participation of physiotherapists in creating awareness regarding their role, lobbying non-governmental organisations and institutions for disability preparedness and ensuring physiotherapy participation during all stages of DM.

Knowledge regarding the role of physiotherapy in DM has increased recommendations for their inclusion in DM in various countries.^1,2,6,9,14^ Such recommendations and measures are required in the Nigerian context, as well as in other African countries. However, inclusion of physiotherapists into the necessary sectors of DM in Nigeria will remain ineffective without appropriate implementation of DM into Nigerian physiotherapy training programmes, providing continuous education programmes on DM, as well as facilitation of specialty clinical practice areas in DM.

The sample for this study did not represent a generalisable population of physiotherapists in Nigeria, which constitutes a major limitation. Considering that this study is in its preliminary phase, it is recommended that follow-up studies integrate larger sample sizes in their design so as to improve on the generalisability of their findings.

### Conclusion

In general, Nigeria-based and trained physiotherapists have moderate knowledge regarding the role of physiotherapy at various stages of DM. However, their involvement in DM activities was reportedly very low. Implementation of policies, involving physiotherapists in the function of the NEMA and at all levels of the Nigerian healthcare care system, will improve the activities and outcomes of DM in the country.

## Data Availability

None.
